# Combined clinical, metabolomic, and polygenic scores for cardiovascular risk prediction

**DOI:** 10.1093/eurheartj/ehaf947

**Published:** 2025-12-15

**Authors:** Scott C Ritchie, Xilin Jiang, Lisa Pennells, Yu Xu, Claire Coffey, Yang Liu, Joel T Gibson, Praveen Surendran, Savita Karthikeyan, Samuel A Lambert, John Danesh, Adam S Butterworth, Angela Wood, Stephen Kaptoge, Emanuele Di Angelantonio, Michael Inouye

**Affiliations:** Cambridge Baker Systems Genomics Initiative, Department of Public Health and Primary Care, University of Cambridge, Papworth Road, Cambridge CB2 0BB, UK; Cambridge Baker Systems Genomics Initiative, Baker Heart & Diabetes Institute, Commercial Road, Melbourne 3004, Victoria, Australia; British Heart Foundation Cardiovascular Epidemiology Unit, Department of Public Health and Primary Care, University of Cambridge, Papworth Road, Cambridge CB2 0BB, UK; Victor Phillip Dahdaleh Heart and Lung Research Institute, University of Cambridge, Papworth Road, Cambridge CB2 0BB, UK; British Heart Foundation Centre of Research Excellence, University of Cambridge, Papworth Road, Cambridge CB2 0BB, UK; Health Data Research UK Cambridge, Wellcome Genome Campus and University of Cambridge, Papworth Road, Cambridge CB2 0BB, UK; Cambridge Baker Systems Genomics Initiative, Department of Public Health and Primary Care, University of Cambridge, Papworth Road, Cambridge CB2 0BB, UK; British Heart Foundation Cardiovascular Epidemiology Unit, Department of Public Health and Primary Care, University of Cambridge, Papworth Road, Cambridge CB2 0BB, UK; Victor Phillip Dahdaleh Heart and Lung Research Institute, University of Cambridge, Papworth Road, Cambridge CB2 0BB, UK; British Heart Foundation Centre of Research Excellence, University of Cambridge, Papworth Road, Cambridge CB2 0BB, UK; Health Data Research UK Cambridge, Wellcome Genome Campus and University of Cambridge, Papworth Road, Cambridge CB2 0BB, UK; Department of Epidemiology, Harvard T.H. Chan School of Public Health, 677 Huntington Ave, Boston, MA 02115, USA; British Heart Foundation Cardiovascular Epidemiology Unit, Department of Public Health and Primary Care, University of Cambridge, Papworth Road, Cambridge CB2 0BB, UK; Victor Phillip Dahdaleh Heart and Lung Research Institute, University of Cambridge, Papworth Road, Cambridge CB2 0BB, UK; Cambridge Baker Systems Genomics Initiative, Department of Public Health and Primary Care, University of Cambridge, Papworth Road, Cambridge CB2 0BB, UK; British Heart Foundation Cardiovascular Epidemiology Unit, Department of Public Health and Primary Care, University of Cambridge, Papworth Road, Cambridge CB2 0BB, UK; Victor Phillip Dahdaleh Heart and Lung Research Institute, University of Cambridge, Papworth Road, Cambridge CB2 0BB, UK; Cambridge Baker Systems Genomics Initiative, Department of Public Health and Primary Care, University of Cambridge, Papworth Road, Cambridge CB2 0BB, UK; British Heart Foundation Cardiovascular Epidemiology Unit, Department of Public Health and Primary Care, University of Cambridge, Papworth Road, Cambridge CB2 0BB, UK; Victor Phillip Dahdaleh Heart and Lung Research Institute, University of Cambridge, Papworth Road, Cambridge CB2 0BB, UK; Cambridge Baker Systems Genomics Initiative, Department of Public Health and Primary Care, University of Cambridge, Papworth Road, Cambridge CB2 0BB, UK; Cambridge Baker Systems Genomics Initiative, Baker Heart & Diabetes Institute, Commercial Road, Melbourne 3004, Victoria, Australia; British Heart Foundation Cardiovascular Epidemiology Unit, Department of Public Health and Primary Care, University of Cambridge, Papworth Road, Cambridge CB2 0BB, UK; Victor Phillip Dahdaleh Heart and Lung Research Institute, University of Cambridge, Papworth Road, Cambridge CB2 0BB, UK; Cambridge Baker Systems Genomics Initiative, Department of Public Health and Primary Care, University of Cambridge, Papworth Road, Cambridge CB2 0BB, UK; British Heart Foundation Cardiovascular Epidemiology Unit, Department of Public Health and Primary Care, University of Cambridge, Papworth Road, Cambridge CB2 0BB, UK; Victor Phillip Dahdaleh Heart and Lung Research Institute, University of Cambridge, Papworth Road, Cambridge CB2 0BB, UK; British Heart Foundation Cardiovascular Epidemiology Unit, Department of Public Health and Primary Care, University of Cambridge, Papworth Road, Cambridge CB2 0BB, UK; British Heart Foundation Centre of Research Excellence, University of Cambridge, Papworth Road, Cambridge CB2 0BB, UK; Rutherford Fund Fellow, Department of Public Health and Primary Care, University of Cambridge, Papworth Road, Cambridge CB2 0BB, UK; British Heart Foundation Cardiovascular Epidemiology Unit, Department of Public Health and Primary Care, University of Cambridge, Papworth Road, Cambridge CB2 0BB, UK; Cambridge Baker Systems Genomics Initiative, Department of Public Health and Primary Care, University of Cambridge, Papworth Road, Cambridge CB2 0BB, UK; British Heart Foundation Cardiovascular Epidemiology Unit, Department of Public Health and Primary Care, University of Cambridge, Papworth Road, Cambridge CB2 0BB, UK; Victor Phillip Dahdaleh Heart and Lung Research Institute, University of Cambridge, Papworth Road, Cambridge CB2 0BB, UK; British Heart Foundation Centre of Research Excellence, University of Cambridge, Papworth Road, Cambridge CB2 0BB, UK; Health Data Research UK Cambridge, Wellcome Genome Campus and University of Cambridge, Papworth Road, Cambridge CB2 0BB, UK; British Heart Foundation Cardiovascular Epidemiology Unit, Department of Public Health and Primary Care, University of Cambridge, Papworth Road, Cambridge CB2 0BB, UK; Victor Phillip Dahdaleh Heart and Lung Research Institute, University of Cambridge, Papworth Road, Cambridge CB2 0BB, UK; British Heart Foundation Centre of Research Excellence, University of Cambridge, Papworth Road, Cambridge CB2 0BB, UK; Health Data Research UK Cambridge, Wellcome Genome Campus and University of Cambridge, Papworth Road, Cambridge CB2 0BB, UK; National Institute for Health and Care Research Blood and Transplant Research Unit in Donor Health and Behaviour, University of Cambridge, Papworth Road, Cambridge CB2 0BB, UK; Department of Human Genetics, Wellcome Sanger Institute, Hinxton CB10 1RQ, UK; British Heart Foundation Cardiovascular Epidemiology Unit, Department of Public Health and Primary Care, University of Cambridge, Papworth Road, Cambridge CB2 0BB, UK; Victor Phillip Dahdaleh Heart and Lung Research Institute, University of Cambridge, Papworth Road, Cambridge CB2 0BB, UK; British Heart Foundation Centre of Research Excellence, University of Cambridge, Papworth Road, Cambridge CB2 0BB, UK; Health Data Research UK Cambridge, Wellcome Genome Campus and University of Cambridge, Papworth Road, Cambridge CB2 0BB, UK; National Institute for Health and Care Research Blood and Transplant Research Unit in Donor Health and Behaviour, University of Cambridge, Papworth Road, Cambridge CB2 0BB, UK; British Heart Foundation Cardiovascular Epidemiology Unit, Department of Public Health and Primary Care, University of Cambridge, Papworth Road, Cambridge CB2 0BB, UK; Victor Phillip Dahdaleh Heart and Lung Research Institute, University of Cambridge, Papworth Road, Cambridge CB2 0BB, UK; British Heart Foundation Centre of Research Excellence, University of Cambridge, Papworth Road, Cambridge CB2 0BB, UK; Health Data Research UK Cambridge, Wellcome Genome Campus and University of Cambridge, Papworth Road, Cambridge CB2 0BB, UK; National Institute for Health and Care Research Blood and Transplant Research Unit in Donor Health and Behaviour, University of Cambridge, Papworth Road, Cambridge CB2 0BB, UK; Cambridge Centre of Artificial Intelligence in Medicine, University of Cambridge, Papworth Road, Cambridge CB2 0BB, UK; British Heart Foundation Cardiovascular Epidemiology Unit, Department of Public Health and Primary Care, University of Cambridge, Papworth Road, Cambridge CB2 0BB, UK; Victor Phillip Dahdaleh Heart and Lung Research Institute, University of Cambridge, Papworth Road, Cambridge CB2 0BB, UK; British Heart Foundation Cardiovascular Epidemiology Unit, Department of Public Health and Primary Care, University of Cambridge, Papworth Road, Cambridge CB2 0BB, UK; Victor Phillip Dahdaleh Heart and Lung Research Institute, University of Cambridge, Papworth Road, Cambridge CB2 0BB, UK; British Heart Foundation Centre of Research Excellence, University of Cambridge, Papworth Road, Cambridge CB2 0BB, UK; Health Data Research UK Cambridge, Wellcome Genome Campus and University of Cambridge, Papworth Road, Cambridge CB2 0BB, UK; National Institute for Health and Care Research Blood and Transplant Research Unit in Donor Health and Behaviour, University of Cambridge, Papworth Road, Cambridge CB2 0BB, UK; Health Data Science Research Centre, Human Technopole, Viale Rita Levi-Montalcini, 20157 Milan, Italy; Cambridge Baker Systems Genomics Initiative, Department of Public Health and Primary Care, University of Cambridge, Papworth Road, Cambridge CB2 0BB, UK; Cambridge Baker Systems Genomics Initiative, Baker Heart & Diabetes Institute, Commercial Road, Melbourne 3004, Victoria, Australia; British Heart Foundation Cardiovascular Epidemiology Unit, Department of Public Health and Primary Care, University of Cambridge, Papworth Road, Cambridge CB2 0BB, UK; Victor Phillip Dahdaleh Heart and Lung Research Institute, University of Cambridge, Papworth Road, Cambridge CB2 0BB, UK; British Heart Foundation Centre of Research Excellence, University of Cambridge, Papworth Road, Cambridge CB2 0BB, UK; Health Data Research UK Cambridge, Wellcome Genome Campus and University of Cambridge, Papworth Road, Cambridge CB2 0BB, UK

**Keywords:** Cardiovascular risk prediction, Biomarker scores, Metabolomics, Polygenic risk scores, Population modelling

## Abstract

**Background and Aims:**

Clinical biomarkers, nuclear magnetic resonance (NMR) metabolomics biomarker scores, and polygenic risk scores (PRS) have shown promise for improving cardiovascular disease (CVD) prediction but have not yet been evaluated in the context of current prediction models (SCORE2) and ESC recommendations for 10-year prediction of fatal and non-fatal CVD.

**Methods:**

NMR metabolomic biomarker scores were constructed and compared to clinical biomarkers, PRS and SCORE2 in 297 463 UK Biobank participants (8919 incident CVD cases) aged 40–69 without previous CVD, diabetes, or lipid-lowering treatment. Improvement in risk discrimination when added to SCORE2 was assessed using Harrel’s C-index. Improvement in risk stratification following ESC guideline risk thresholds was assessed using categorical net reclassification. Population modelling was subsequently applied to estimate the impact on CVD prevention if applied at scale.

**Results:**

Risk discrimination provided by SCORE2 (C-index: 0.719) improved when 11 clinical biomarkers (ΔC-index: 0.014 [0.012–0.015]), NMR metabolomic biomarker scores (ΔC-index: 0.010 [0.009–0.012]) and PRSs (ΔC-index 0.009; [0.008–0.011]) were added individually. The combination of 11 clinical biomarkers, NMR metabolomic biomarker scores, and PRSs yielded the largest improvement risk discrimination, with ΔC-index 0.024 (0.022–0.027). Concomitant improvements in risk stratification were observed in categorical net reclassification index, with net case reclassification of 16.66% (15.50%–17.81%). Modelling suggested that addition of these biomarkers to SCORE2 for targeted risk reclassification would increase the number of CVD events prevented per 100 000 screened from 229 to 413 (ΔCVD_prevented_: 184 [174–194]) while essentially maintaining the number of statins prescribed per CVD event prevented.

**Conclusions:**

Combining NMR metabolomic, polygenic, and clinical biomarkers with SCORE2 enhanced prediction of first-onset CVD and could have substantial population health benefit if applied at scale.

## Structured Graphical Abstract

**Structured Graphical Abstract:**
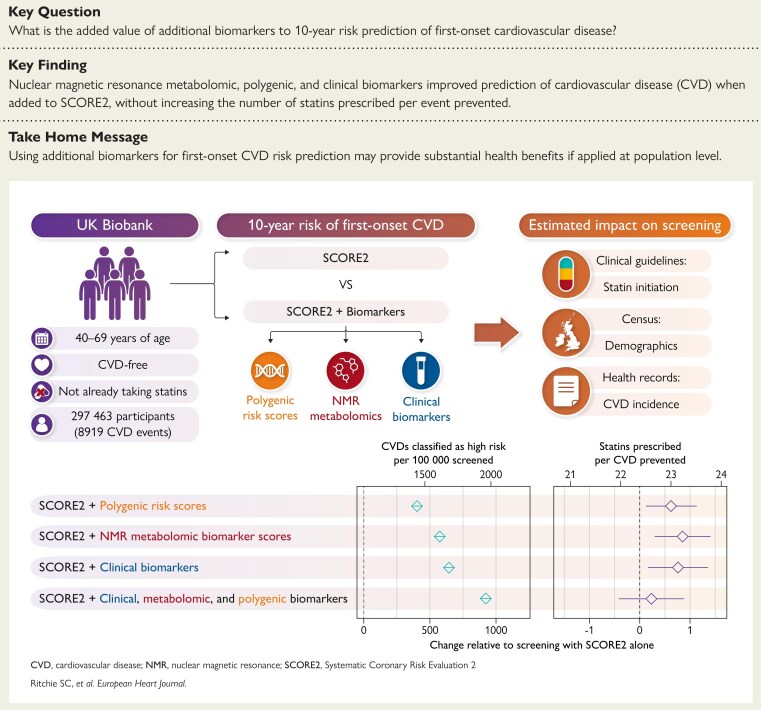
Study schematic followed by graph showing key findings of how addition of NMR metabolomic, polygenic risk scores, and clinical biomarkers could have population health benefit if applied at scale. The plot on the left shows how the number of future CVD events within 10-years correctly classified as high-risk changes when using biomarkers for targeted re-screening of people classified as medium risk with SCORE2. Numbers on the axis above the plot show how the total number of future CVD events per 100 000 screened changes, while the numbers on the bottom show the change relative to population-wide screening with SCORE2. The plot on the right shows how the number of statins prescribed per CVD event prevented remains unchanged when adding biomarkers to SCORE2. Numbers above the plot show the total number of statins prescribed per CVD event prevented, assuming one in five events are prevented by statin initiation in the high-risk group.

**See the editorial comment for this article ‘Risk prediction in primary prevention: personalized, precise but practicable?’, by H. Schunkert, https://doi.org/10.1093/eurheartj/ehaf946.**

Translational perspective Risk modifiers for refining 10-year prediction of cardiovascular disease (CVD) have gained considerable interest in the last decade and are advocated by the 2021 ESC Guidelines for primary CVD prevention in clinical practice. This study performed the largest population health assessment to date of addition of new biomarkers to the current ESC-recommended prediction model (SCORE2). It shows addition of further clinical biomarkers, NMR metabolomic biomarkers, and polygenic risk scores can enhance prediction of first-onset CVD. It supports serious consideration of additional biomarkers as risk modifiers by the ESC and may impact future position statements and guidelines for CVD prediction and prevention.

## Introduction

Circulating biomarkers play a central role in cardiovascular disease (CVD) risk scores recommended by clinical guidelines to identify high-risk individuals for primary CVD prevention.^1–3^ Total cholesterol and high-density lipoprotein (HDL) cholesterol are routinely measured and used alongside demographic and lifestyle risk factors to assess 10-year risk of CVD using risk scores such as SCORE2^4^. However, clinical guidelines have long-recognized the need to identify new risk factors to improve primary prevention.^1–3^ Depending on the risk algorithm used and the definition of the treatment threshold, it is estimated that between 40%–86% of first-onset CVD events occur in otherwise apparently healthy adults who would be classified as low-risk using conventional risk factors.^5^ Efforts to improve CVD risk prediction models have considered additional circulating biomarkers,^6^ such as C-reactive protein (CRP),^7,8^ as well as incorporating genetic biomarkers in the form of polygenic risk scores (PRSs).^9–13^ While PRSs have shown potential to enhance CVD risk screening,^14–19^ addition of individual established CVD biomarkers have thus far shown limited overall incremental benefits.^20–22^

High-throughput nuclear magnetic resonance (NMR) spectroscopy has enabled rapid and simultaneous quantification of metabolomic biomarkers from a single human blood plasma sample.^23,24^ These include cholesterols and other lipids in lipoprotein sub-fractions, fatty acids, ketone bodies, amino acids, glycolysis metabolites and inflammation. NMR metabolic biomarker data has been quantified in numerous cohorts over the last decade, helping derive new insights into the genetic determinants, molecular pathogenesis, and epidemiology of CVD.^25^ Several studies have investigated the utility of biomarker combinations from NMR platforms to improve prediction of first-onset CVD^26–29^; however, they have focused on multi-disease prediction, used outdated clinical risk prediction scores, and have not investigated improvements relative to clinically relevant guideline-recommended risk thresholds.

Here, we utilize clinical biochemistry assay data and NMR biomarker data in UK Biobank to assess whether these biomarkers can improve 10-year CVD risk prediction in apparently healthy adults when added to the SCORE2 risk model, which is recommended by the European Society of Cardiology (ESC) 2021 guidelines for primary prevention of CVD.^3^ We further assess whether incremental improvements in CVD risk prediction are meaningful at ESC 2021 recommended risk thresholds for treatment consideration.^3^ In addition, we compared the improvement in risk prediction provided by these biomarkers to that provided by PRS^15^ and assessed the combined performance of metabolomic, PRS and established clinical biomarkers. Finally, we modelled the potential public health benefits for CVD prevention if applied to the UK primary care population according to the ESC 2021 guidelines for statin initiation.

## Methods

### Study cohort

**Figure 1:**
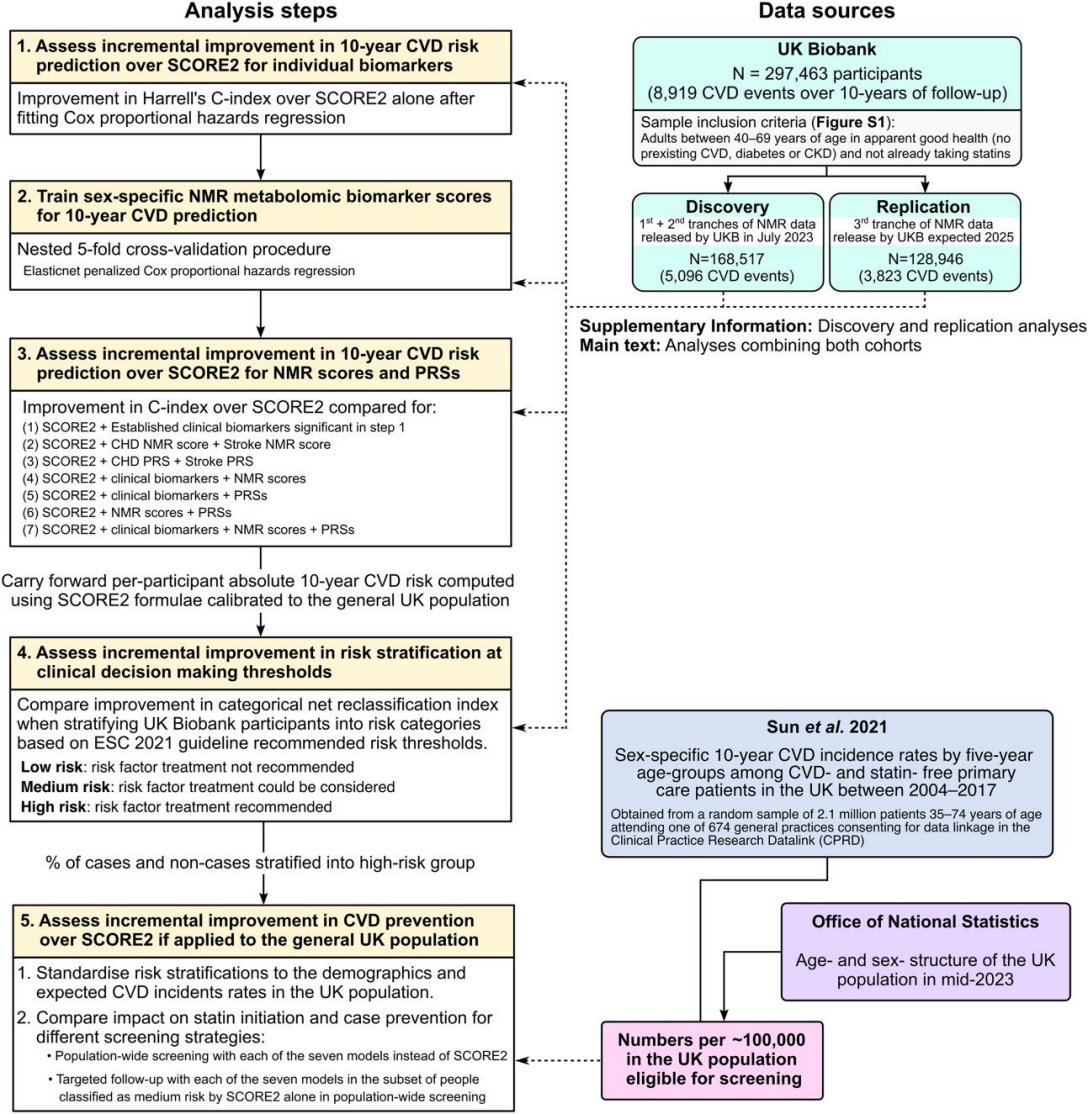
Study design. For details on sample inclusion and exclusion criteria when defining study eligibility for UK Biobank participants see Supplementary data online, *Figure S1*. Details on the NMR metabolomic biomarker score training procedure are provided in the Supplementary Methods

Modelling was performed in UK Biobank^30,31^ participants who were eligible for primary 10-year CVD risk assessment with SCORE2^3^: those who were 40–69 years of age at baseline assessment and had no prior history of established atherosclerotic cardiovascular disease, diabetes mellitus, chronic kidney disease, or familial hypercholesterolemia. Prevalent atherosclerotic cardiovascular disease included acute myocardial infarction, acute coronary syndromes, stroke, transient ischaemic attack, peripheral arterial disease, and history of revascularization procedures. Participants already taking lipid-lowering medications were also excluded as the primary goal of the study was to improve identification of apparently healthy adults who would most benefit from lipid-lowering medications for primary prevention of CVD. Participants were also excluded if they did not consent to electronic health record linkage, had incomplete data on SCORE2 risk factors, were ineligible for PRS assessment, or failed NMR biomarker quality control. Further information on the sample exclusion criteria is detailed in the Supplementary Methods. Of the 502 207 participants enrolled in UK Biobank consenting to electronic health record linkage, 297 463 participants met the inclusion criteria for this study (see Supplementary data online, *Figure S1*). A schematic of the overall study is given in *Figure 1*.

### Cardiovascular disease endpoint

The primary endpoint of the study was first-onset CVD within 10-years. CVD events were extracted from linked hospital episode statistics and death registry data (see Supplementary Methods) following the definition used by the SCORE2 working group and ESC Cardiovascular risk collaboration.^4^ This included International Classification of Diseases (ICD) codes covering: fatal hypertensive disease (ICD-10 codes I10–I16), fatal ischaemic heart disease (ICD-10 codes I20–I25), fatal arrhythmias or heart failure (ICD-10 codes I46–I52, excluding I51.4), fatal cerebrovascular disease (ICD-10 codes I60–I69, excluding I60, I62, I67.1, I68.2, and I67.1), fatal atherosclerosis or abdominal aortic aneurysm (ICD-10 codes I70–I73), sudden death and death within 24 h of symptom onset (ICD-10 codes R96.0 and R96.1), non-fatal myocardial infarction (ICD-10 I21–I23), and non-fatal stroke (ICD-10 codes I60–I69, excluding I60, I62, I67.1, I68.2, and I67.1). During the 2 897 497 person-years at risk (median [5th, 95th percentile] follow-up of 10.0 [8.4–10.0] years), 8919 CVD cases were recorded. Baseline cohort characteristics are detailed in *Table 1*.

**Table 1:** Cohort characteristics

Baseline characteristics	Males	Females	Total
Number of participants	127 269	170 194	297 463
Age (years), mean (SD)	55 (8.2)	55 (7.9)	55 (8.0)
Cardiovascular risk factors			
Current smokers, *n* (%)	15 561 (12.2%)	14 510 (8.5%)	30 071 (10.1%)
SBP (mmHg), mean (SD)	140 (17)	134 (19)	137 (19)
Total cholesterol (mmol/L), mean (SD)	5.8 (1.0)	6.0 (1.1)	5.9 (1.1)
HDL cholesterol (mmol/L), mean (SD)	1.3 (0.3)	1.6 (0.4)	1.5 (0.4)
SCORE2 10-year CVD risk (%), mean (SD)	5.6% (3.0%)	3.3% (2.2%)	4.3% (2.8%)
Incident CVD within 10 years of follow-up			
Events, *n* (%)	5633 (4.4%)	3286 (1.9%)	8919 (3.0%)
Years to first event, median (IQR)	6.1 (3.5, 8.2)	6.6 (4.1, 8.6)	6.3 (3.7, 8.4)
Fatal events, *n* (%)	1140 (20.2%)	514 (15.6%)	1654 (18.5%)
Event is primary cause of hospitalization or death, *n* (% of events)	4312 (76.5%)	2176 (66.2%)	6488 (72.7%)
Non-CVD mortality within 10 years of follow-up			
Events, *n* (%)	4078 (3.2%)	3945 (2.3%)	8023 (2.7%)
Years to fatal event, median (IQR)	6.1 (3.5, 8.2)	6.2 (3.8, 8.2)	6.2 (3.7, 8.2)
Lost to follow-up before 10 years			
Events, *n* (%)	2649 (2.1%)	3597 (2.1%)	6246 (2.1%)
Maximum follow-up in Welsh hospital records, *n* (% lost to follow-up)	2337 (88.2%)	3237 (90.0%)	5574 (89.2%)
Years until end of Welsh hospital record linkage, median (IQR)	9.8 (8.5, 9.9)	9.8 (8.6, 9.9)	9.8 (8.6, 9.9)
Other reason (e.g. left UK), *n* (% lost to follow-up)	312 (11.8%)	360 (10.0%)	672 (10.8%)
Years until lost to follow-up, median (IQR)	4.6 (3.5, 5.4)	4.3 (3.0, 5.3)	4.4 (3.2, 5.3)

SBP, systolic blood pressure; HDL, High-density lipoprotein; LDL, Low density lipoprotein; SD, standard deviations; IQR, interquartile range.

### Study design

Study participants were further split into a discovery and replication cohort for all analyses based on the timing of the availability of NMR metabolomic data (*Figure 1*, Supplementary data online, *Figure S1*). The discovery cohort comprised the subset of 167 517 eligible study participants (5096 CVD cases) for whom NMR metabolomic data was made publicly available by UK Biobank in July 2023. The replication cohort comprised the remaining 128 946 participants (3823 CVD cases) for whom we had early access to their NMR metabolomic data under UK Biobank project #30418 from Q3 of 2024 onwards. Allocation of participants to measurement tranches was randomized by UK Biobank with respect to phenotypes. Characteristics of the discovery and replication cohorts were similar (see Supplementary data online, *Table S1*). Results presented in the main text are from pooled analyses across the full study cohort, with key differences from discovery and replication cohorts noted. Results in the discovery and replication cohorts for all analyses are presented in the corresponding Supplementary data online, *Figures* and *Tables*.

### Incremental value in 10-year CVD risk prediction for individual biomarkers

Improvements in 10-year CVD risk discrimination were assessed by changes in Harrell's C-index (ΔC-index) beyond SCORE2 alone. SCORE2 was computed from age, sex, smoking status, systolic blood pressure (SBP), total cholesterol, and high-density lipoprotein (HDL) cholesterol using established coefficients.^4^ The total and HDL cholesterol measures used were those quantified by clinical biochemistry assays (Beckman Coulter Inc).^32^ Further details on measurement assays, data quantification, SCORE2 computation, and statistical modelling are provided in the Supplementary Methods.

Improvements in 10-year CVD risk discrimination were assessed for each of the 249 NMR metabolomic biomarkers^33,34^ (see Supplementary data online, *Table S2*) and 28 clinical chemistry biomarkers^32^ (see Supplementary data online, *Table S3*) assayed in UK Biobank. In the discovery cohort, sex-stratified Cox proportional hazards regressions were fit for 10-year CVD risk for each biomarker, with the biomarker as an independent variable and SCORE2 as an offset term. In the replication cohort, the model fit for each biomarker was predicted using hazard ratios obtained from the discovery cohort. Predicted model fits were pooled across the discovery and replication cohorts for analyses presented in the main text. *P*-values were corrected for multiple testing across the 277 tested biomarkers using Benjamini–Hochberg false discovery rate (FDR) correction. Further details are given in the Supplementary Methods.

### Incremental value in 10-year CVD risk prediction for biomarker combinations and PRSs

To assess whether combinations of biomarkers could improve 10-year CVD risk discrimination we assessed (1) a model combining SCORE2 with NMR metabolomic biomarker scores trained using machine learning models to maximize CVD prediction (see Supplementary Methods), and (2) a multivariable model of SCORE2 + clinical biochemistry biomarkers passing FDR significance in assessment of individual biomarkers above. Different modelling approaches were taken as collinearity prevented joint modelling of FDR-significant NMR metabolomic biomarkers, and conversely, clinical biomarker scores trained using machine learning models yielded substantially smaller improvements in CVD prediction compared to multivariable modelling of the FDR-significant clinical biomarkers (data not shown).

Models combining SCORE2 with NMR metabolomic biomarker scores and/or clinical biomarkers were additionally compared to and combined with PRSs for coronary heart disease^10^ (Polygenic Score Catalog^35^ accession PGS000018) and ischaemic stroke^11^ (PGS000039) which have previously been shown to enhance 10-year CVD risk prediction when added alongside conventional risk factors.^15^ Notably, PRSs capture inherited lifetime risk due to genetics,^10–12^ whereas biomarkers capture part of the dynamic component of risk conferred by lifestyle and environment.^26^

In total, we compared seven models to SCORE2: (1) SCORE2 + clinical biomarkers, (2) SCORE2 + NMR metabolomic biomarker scores, (3) SCORE2 + PRSs, (4) SCORE2 + clinical biomarkers + PRSs, (5) SCORE2 + NMR metabolomic biomarker scores + PRSs, (6) SCORE2 + NMR metabolomic biomarker scores + clinical biomarkers, and (7) SCORE2 + NMR metabolomic biomarker scores + clinical biomarkers + PRSs. Further details on the multivariable model fits are given in the Supplementary Methods. Improvements in 10-year CVD risk discrimination were assessed as described above, with multivariable models fit in the discovery cohort and predicted in the replication cohort.

### Incremental value in 10-year CVD risk stratification at risk thresholds used for clinical decision making

Categorical net reclassification improvement (NRI) analysis^36,37^ was used to assess the incremental value of the seven multivariable models over SCORE2 for stratifying individuals based on ESC 2021 recommended risk thresholds for treatment consideration.^3^ For each model, study participants <50 years of age were stratified into low, medium, and high-risk groups if their predicted absolute risk was <2.5%, <7.5%, and ≥7.5% respectively, and study participants ≥50 years of age were stratified at <5%, <10%, ≥10% respectively. Categorical NRI analysis was used to assess (1) the percentage of incident CVD cases correctly reclassified from a lower risk group into higher risk group, and (2) the percentage of non-cases correctly reclassified from a higher risk group into a lower risk group. Further details on the computation of absolute risks for each model and the NRI analysis are provided in the Supplementary Methods.

### Incremental value for CVD prevention for population-wide and targeted screening

Two different screening strategies were assessed for incremental benefits for primary prevention of CVD if applied to the UK primary care population eligible for screening: (1) population-wide screening, in which all people eligible for screening were assessed with each model; and (2) targeted screening, in which people were first stratified into low, medium, and high-risk groups using SCORE2 alone, then those allocated to the medium-risk group were re-assessed using the models adding NMR scores, clinical biomarkers, and/or PRSs to SCORE2. For targeted re-screening we compared each model to (1) population-wide screening with SCORE2, and (2) instead classifying the same number of people as high-risk prioritized by SCORE2 to provide a fair comparison, as targeted risk reclassification is inherently biased towards improved outcomes.^38^ Further details are provided in the Supplementary Methods.

Incremental improvements in primary CVD prevention for each alternative model were assessed by differences from SCORE2 alone per 100 000 screened in (1) the number classified as high risk (ΔN_high-risk_); (2) the number of future CVD cases amongst the high-risk group (ΔCVD_high-risk_); (3) the number of future CVD events expected to be prevented by initiation of statins in the high-risk group (ΔCVD_prevented_); (4) the number needed to screen to prevent one CVD event (ΔNNS); and the number of statins prescribed per CVD event prevented (ΔNNT). The impact of statin initiation was modelled as preventing one in five incident CVD events.^39^

To account for the healthy ascertainment bias of UK Biobank,^30^ numbers of CVD cases and non-cases stratified into each risk group by each model were standardized to the demographics and expected CVD incidence rates of the UK primary care population eligible for screening. Demographic data on age and sex distributions of the general UK population were obtained from mid-2023 estimates published by the Office for National Statistics^40^ and 10-year CVD incidence rates were obtained from previously published estimates^15^ extracted from CVD- and statin- free primary care patients. Standard errors, 95% confidence intervals, and *P*-values were obtained in a bootstrap procedure with 1000 bootstraps. Further details are provided in the Supplementary Methods.

### Sensitivity analyses

Throughout the study we compared results to those obtained from analyses performed in males and females separately, using QRISK3 instead of SCORE2 as the risk score,^41^ and using narrower set of criteria for identifying incident CVD events. The more narrowly defined CVD endpoint was defined to include only incident myocardial infarction (ICD-10 codes I21 and I22), fatal coronary heart disease (ICD-10 codes I20–I25), and fatal cerebrovascular events (ICD-10 codes I60–I69 or F01). Further details on QRISK3 and its computation are given in the Supplementary Methods.

## Results

### Incremental value in 10-year CVD risk prediction for individual biomarkers

**Figure 2:**
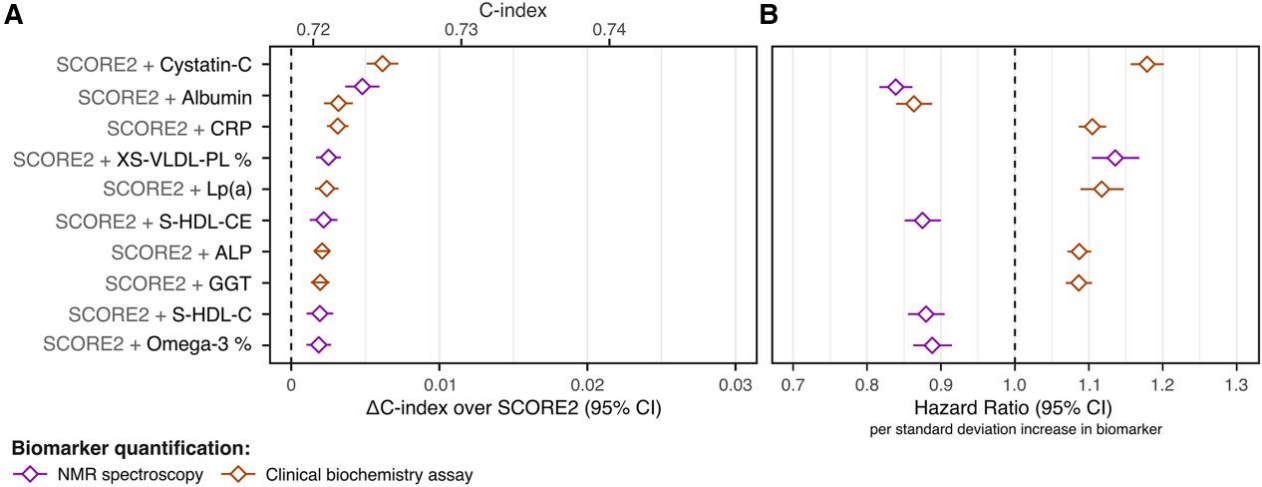
Incremental 10-year CVD risk discrimination over SCORE2 for individual biomarkers. (*A*) Change in C-index (ΔC-index) relative to SCORE2 for the top 10 biomarkers by ΔC-index. The ΔC-index were assessed for each biomarker and quantification method separately (277 tests total; Supplementary data online, *Table S4*) in the subset of the 297 463 study participants (8919 CVD cases) with non-missing biomarker concentrations for each test. Among the top 10 biomarker shown, only albumin concentrations were quantified by both clinical biochemistry assays (orange) and high-throughput NMR spectroscopy (purple). (*B*) Hazard ratios per standard deviation increase in the respective biomarker concentration in the sex-stratified Cox proportional hazards model fit with SCORE2 as an offset term in the 168 517 study participants in the discovery cohort, which were used to predict the SCORE2 + biomarker model fit in the replication cohort (Methods)

Across all UK Biobank participants eligible for CVD screening for primary prevention (*n* = 297 463) the sex-stratified C-index for SCORE2 alone was 0.719 (95% confidence interval [CI]: 0.714, 0.723) for predicting first-onset CVD within 10 years of follow-up (8919 cases). When testing improvements in CVD risk discrimination for each of the 249 NMR biomarkers (see Supplementary data online, *Table S2*) and 28 clinical chemistry biomarkers individually (see Supplementary data online, *Table S3*) we observed statistically significant improvement in C-index for 66 of the 277 biomarkers (see Supplementary data online, *Table S4*) based on a false discovery rate (FDR) adjusted *P*-value < .05. Improvements in sex-stratified C-index over SCORE2 (ΔC-index) were observed with addition of biomarkers (*Figure 2A*). The largest ΔC-index observed for any biomarker was with addition of cystatin-C measured by clinical biochemistry assay (*Figure 2A*), with ΔC-index of 0.006 (95% CI: 0.005, 0.007; *P*-value: 2 × 10^−29^; FDR: 6 × 10^−27^; Supplementary data online, *Table S4*). The largest ΔC-index observed for any of the 249 NMR biomarkers was with addition of albumin (*Figure 2A*), with ΔC-index of 0.005 (95% CI: 0.004, 0.006; *P*-value: 4 × 10^−16^; FDR: 4 × 10^−14^; Supplementary data online, *Table S4*).

Results were similar when analysing the discovery (*n* = 167 517; 5096 CVD cases) and replication (*n* = 128 946; 3823 CVD cases) cohorts separately (see Supplementary data online, *Figure S2A*, Supplementary data online, *Table S5*). Results were also similar when analysing males (*n* = 127 269; 5633 CVD cases) and females (*n* = 170 194; 3286 CVD cases) separately, with ΔC-index estimates consistent for the strongest biomarkers (see Supplementary data online, *Table S2B*, *Table S5*). Results were also similar when using a more narrowly defined CVD definition (see Supplementary data online, *Figure S2C*, Supplementary data online, *Table S5*). Improvements in ΔC-index from individual biomarkers were smaller when using QRISK3 instead of SCORE2 as the conventional risk score (see Supplementary data online, *Figure S2D*) with 16 of 277 biomarkers significantly increasing C-index at FDR < 0.05 (see Supplementary data online, *Table S5*).

### Addition of multivariable biomarker models to SCORE2

**Figure 3:**
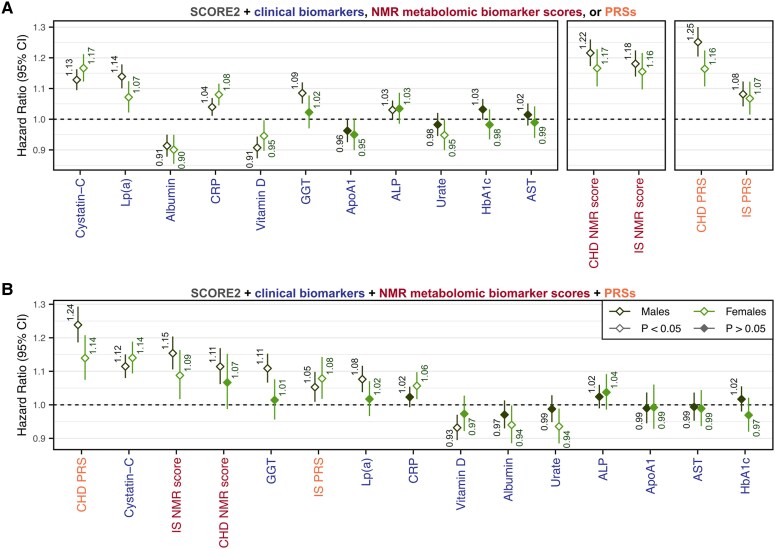
Multivariable models of clinical biomarkers, NMR scores, and PRSs with SCORE2. (*A*) Multivariable models of (1) SCORE2 + clinical biomarkers, (2) SCORE2 + NMR metabolomic biomarker scores, and (3) SCORE2 + PRSs. (*B*) Multivariable model of SCORE2 + clinical biomarkers + NMR metabolomic biomarker scores + PRSs. (*A* and *B*) Each model was fit as a sex-specific Cox proportional hazards regression with SCORE2 as an offset term in the discovery cohort (n = 168 517; 5096 CVD cases). Hazard ratios are per standard deviation increase in the respective biomarker, NMR score, or PRS and are detailed in Supplementary data online, *Table S7*. Log hazard ratios were used to predict the sex-specific models in the replication cohort (see Supplementary Methods). The 11 clinical biomarkers used were those that had FDR-significant ΔC-index over SCORE2 alone when testing individual biomarkers (see Supplementary data online, *Table S4*). Details on the NMR scores are given in Supplementary data online, *Table S6* and the Supplementary Methods. CHD, coronary heart disease; IS, ischaemic stroke; Lp(a), Lipoprotein(a); CRP, C-reactive protein; GGT, Gamma glutamyltransferase; ApoA1, Apolipoprotein A1; ALP, Alkaline phosphatase; HbA1c, Glycated haemoglobin; AST, Aspartate aminotransferase

In total, 11 FDR-significant clinical chemistry biomarkers were carried forward for joint modelling with SCORE2 (see Supplementary data online, *Table S4*): cystatin-C, CRP, alkaline phosphatase (ALP), albumin, gamma glutamyltransferase (GGT), lipoprotein(a) [Lp(a)], aspartate aminotransferase (AST), glycated haemoglobin (HbA1c), urate, vitamin D, and apolipoprotein A1 (ApoA1). NMR metabolic biomarkers were combined into NMR biomarker scores for predicting coronary heart disease and ischaemic stroke each comprising the 106 fundamental metabolic measures on the NMR panel (see Supplementary data online, *Table S6*, Supplementary Methods). Relative contributions of each clinical biomarker, NMR score, and PRS to 10-year CVD prediction when added to SCORE2 (see Supplementary data online, *Table S7*, Supplementary Methods) are shown in *Figure 3*.

In multivariable models of SCORE2 + clinical biomarkers, the biomarker with the strongest association with 10-year CVD risk was cystatin-C in both males (Hazard Ratio: 1.13, 95% CI: 1.10–1.16, *P*-value: 5 × 10^−15^) and females (HR: 1.17, 95% CI: 1.12–1.21, *P*-value: 3 × 10^−15^), followed by Lp(a) in males (HR: 1.14, 95% CI: 1.10–1.18, *P*-value: 1 × 10^−13^) and CRP in females (HR: 1.08, 95% CI: 1.05–1.12, *P*-value: 4 × 10^−6^) (*Figure 3A*, Supplementary data online, *Table S7*). When jointly modelled together, hazard ratios for clinical biomarkers, NMR scores, and PRSs were attenuated but in most cases remained statistically significant (*Figure 3B*, Supplementary data online, *Table S7*), indicating that these biomarkers largely captured independent components of 10-year CVD risk. Results were similar when fitting multivariable models with the individual risk factors comprising SCORE2 (see Supplementary data online, *Figure S13*, Supplementary data online, *Table S21*).

### Incremental value in 10-year CVD risk prediction for biomarker scores

**Figure 4:**
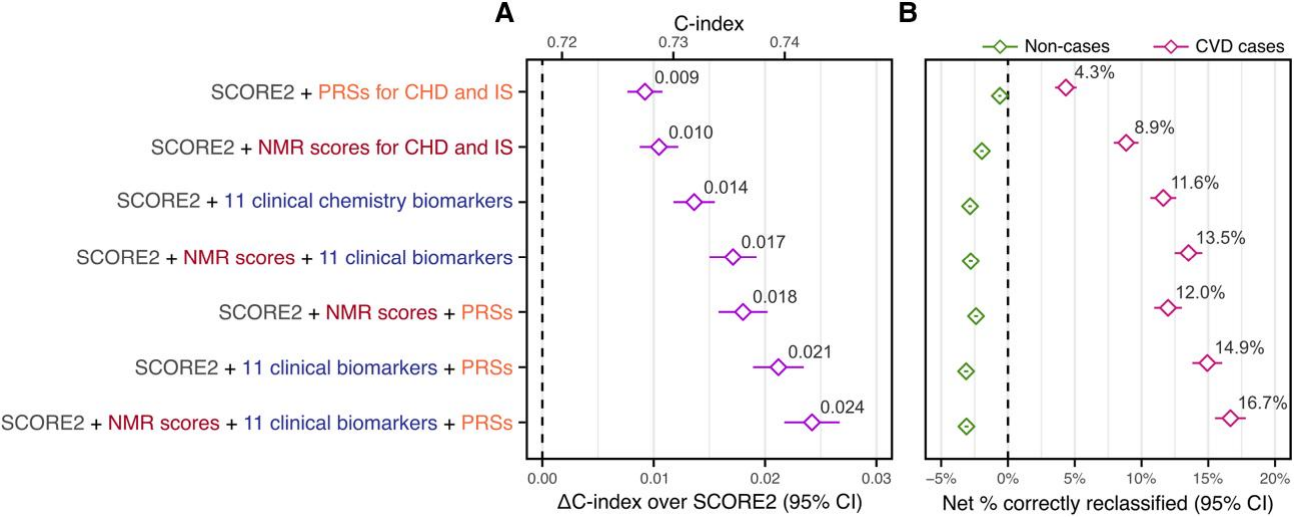
Incremental value in 10-year CVD risk prediction for biomarker combinations and PRSs. (*A*) Incremental improvement in 10-year CVD risk discrimination over SCORE2 alone (ΔC-index) in the 297 463 study participants (8919 CVD cases) when adding NMR scores, clinical biomarkers, and/or PRSs to SCORE2. NMR scores for coronary heart disease (CHD) and ischaemic stroke (IS) each comprised the 106 non-derived biomarkers on the NMR platform and are detailed in Supplementary data online, *Table S6* and the Supplementary Methods. The 11 clinical biochemistry biomarkers added to SCORE2 were cystatin-C, C-reactive protein, alkaline phosphatase, albumin, gamma glutamyltransferase, lipoprotein(a), aspartate aminotransferase, glycated haemoglobin (HbA1c), urate, vitamin D, and Apolipoprotein A1 (see Supplementary data online, *Table S7*). The PRSs for CHD and IS added to SCORE2 were those published in the PGS Catalog with accession numbers PGS000018 and PGS000039 respectively. Details on the multivariable fits for each of the seven models are provided in Supplementary data online, *Table S7* and the Supplementary Methods. C-indices, ΔC-indices, and 95% confidence intervals for each model are provided in Supplementary data online, *Table S9*. (*B*) Categorical net reclassification improvement (NRI) index relative to SCORE2 when stratifying the 297 463 study participants (8919 CVD cases) into low-, medium-, and high-risk categories based on the absolute 10-year CVD risk predicted by each model and ESC 2021 recommended risk thresholds for treatment consideration (Methods). Net % correctly reclassified: net % of CVD cases that were correctly reclassified into a higher risk category (pink) or net % of non-cases that were correctly reclassified into a lower risk category (green) when comparing the given model to SCORE2. Categorical NRI details are provided in Supplementary data online, *Table S11*. Numbers allocated to each risk category by each model are detailed in Supplementary data online, *Table S12*

When adding PRSs, NMR metabolomic biomarker scores, or clinical biomarkers to SCORE2 we observed statistically significant improvements in ΔC-index of 0.009 (95% CI: 0.008, 0.011; *P*-value: 1 × 10^−30^), 0.010 (95% CI: 0.009, 0.012; *P*-value: 1 × 10^−32^), and 0.014 (95% CI: 0.012, 0.015; *P*-value: 7 × 10^−47^) respectively (*Figure 4A*, Supplementary data online, *Table S9*). Improvement in risk discrimination was greatest with addition of PRSs, NMR scores, and clinical biomarkers combined (*Figure 4A*), with ΔC-index of 0.024 (95% CI: 0.022, 0.027; *P*-value: 3 × 10^−81^) (see Supplementary data online, *Table S9*)—an 11.1% gain in C-index relative to SCORE2 alone—for a total absolute C-index of 0.743 compared to 0.719 for SCORE2 alone.

Results were similar when analysing the discovery and replication cohorts separately (see Supplementary data online, *Figure S4A*, Supplementary data online, *Table S10*) with slightly higher ΔC-index estimates in the discovery cohort as expected. Results were also broadly similar when analysing males and females separately, with stronger improvements in ΔC-index in males than in females for models incorporating PRSs (see Supplementary data online, *Figure S4B*, Supplementary data online, *Table S10*). Results were also similar when using a more narrowly defined CVD definition (see Supplementary data online, *Figure S4C*, Supplementary data online, *Table S10*) and when using QRISK3 instead of SCORE2 as the conventional risk score (see Supplementary data online, *Figure S4D*, Supplementary data online, *Table S10*). Notably attenuation of ΔC-index when using QRISK3 was less pronounced when combining information across multiple biomarkers (see Supplementary data online, *Figure S4D*) compared to the attenuation observed when assessing biomarkers separately (see Supplementary data online, *Figure S2D*).

### Incremental value in 10-year CVD risk stratification at risk thresholds used for clinical decision making

Statistically significant improvement in risk stratification over SCORE2 among incident CVD cases was observed for all seven alternative models tested (*Figure 4B*, Supplementary data online, *Tables S11* and *S12*), when using ESC 2021 recommended risk thresholds for treatment consideration.^3^ Improvements in case classification when adding either the NMR biomarker scores or the 11 clinical biomarkers to SCORE2 were more than twice as strong as those from PRSs. We observed a net case reclassification rate of 8.85% (95% CI: 7.90%, 9.80%; *P*-value: 8 × 10^−74^) with addition of NMR scores and 11.63% (95% CI: 10.62%, 12.64%; *P*-value: 5 × 10^−112^) with addition of the 11 clinical biomarkers, compared to 4.33% (95% CI: 3.52%, 5.15%; *P*-value: 2 × 10^−25^) with addition of PRSs (see Supplementary data online, *Table S11*). Improvements in case classification were strongest with addition of PRSs, NMR scores, and clinical biomarkers combined (*Figure 4B*), with a net case reclassification rate of 16.66% (95% CI: 15.50%, 17.81%; *P*-value: 1 × 10^−175^) (see Supplementary data online, *Table S11*).

A statistically significant, inappropriate reclassification for non-cases was also observed for all seven alternative models (*Figure 4B*, Supplementary data online, *Table S11*). The net reclassification rate for non-cases was −1.99% (95% CI: −2.16%, −1.81%; *P*-value: 3 × 10^−199^) with addition of NMR scores, −2.84% (95% CI: −2.98%, −2.70%; *P*-value: < 3 × 10^−308^) with addition of the 11 clinical biomarkers, −0.61% (95% CI: −0.73%, −0.49%; *P*-value: 2 × 10^−23^) with the addition of PRSs, and −3.11% (95% CI: −3.26%, −2.95%; *P*-value: < 3 × 10^−308^) with addition of PRSs, NMR scores, and clinical biomarkers combined (see Supplementary data online, *Table S11*).

Results were similar when analysing the discovery and replication cohorts separately (see Supplementary data online, *Figure S5A*, Supplementary data online, *Table S13* and *S14*), when analysing males and females separately (see Supplementary data online, *Figure S5B*), and when using a more narrowly defined CVD definition (see Supplementary data online, *Figure S5C*). Notably, inappropriate net reclassification of non-cases in males was approximately half that of the inappropriate net reclassification of non-cases observed in females (see Supplementary data online, *Figure S5B*, Supplementary data online, *Table S13*). Improvements in net case reclassification were strongly attenuated, although remained statistically significant, when adding NMR scores, 11 clinical biomarkers, and/or PRSs to QRISK3 and using the 10% risk threshold recommended alongside QRISK3 by the UK's National Institute for Health and Care Excellence (NICE) 2023 guidelines for cardiovascular risk assessment and reduction^41^ (see Supplementary data online, *Figure S5D*, Supplementary data online, *Table S13*). Notably, there was a strong increase in the proportion of participants classified as high risk by QRISK3 alone with increasing age (e.g. >99% of males >65 years of age; Supplementary data online, *Table S15*), suggesting the observed attenuation was a consequence of the dominating influence of age on treatment consideration when following the NICE 2023 guidelines with QRISK3.

### Incremental value for CVD prevention for population-wide and targeted screening

**Figure 5:**
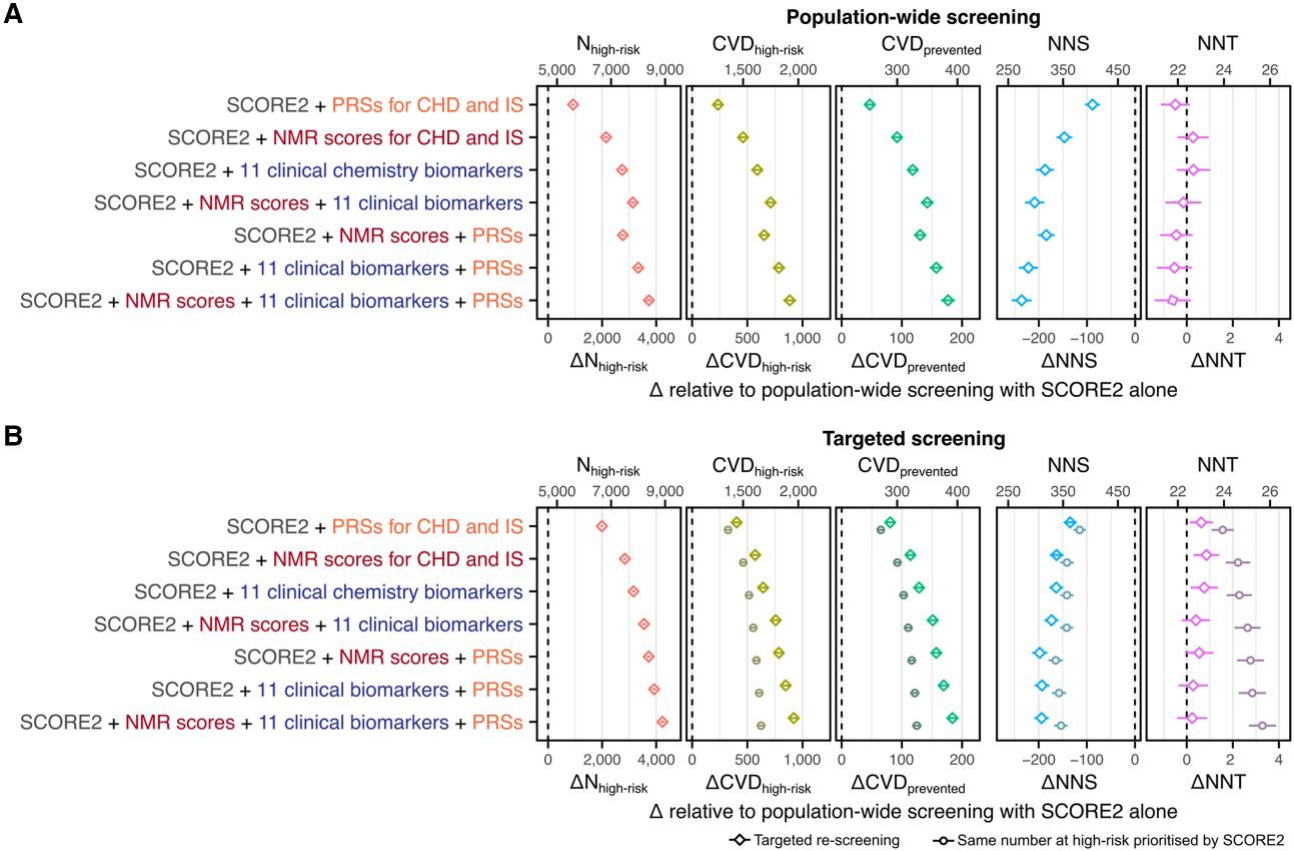
Incremental value for CVD prevention per 100 000 in the UK eligible for screening. (*A*) Incremental value for primary prevention of CVD per 100 000 in the UK eligible for screening when adding NMR scores, clinical biomarkers, and/or PRSs to SCORE2. Per 100 000 the number of CVD events expected within 10 years was 6686 (see Supplementary Methods). (*B*) Incremental value for primary prevention of CVD when using NMR scores, clinical biomarkers, and/or PRSs for targeted risk reclassification in people classified as medium risk by SCORE2 alone (36 054 per 100 000; 3765 CVD events) compared to classifying the same number of people as high-risk prioritized by SCORE2. (*A* and *B*) ΔN_high-risk_: change in the number per 100 000 classified as high-risk relative to SCORE2 alone. ΔCVD_high-risk_: change in the number of incident CVD events classified as high-risk per 100 000 screened. ΔCVD_prevented_: change in the number of CVD events expected to be prevented over 10 years due to statin initiation in the high-risk group. ΔNNS: change in number needed to screen to prevent one CVD event. ΔNNT: change in the number of statins prescribed per CVD event prevented. Estimates were derived by standardizing risk stratification in the study cohort to the demographics and expected CVD incidence rates of the UK primary care population eligible for screening (Methods). 95% confidence intervals were estimated via a bootstrap sampling procedure with 1000 bootstraps (Methods). Point estimates and 95% confidence intervals are detailed in Supplementary data online, *Table S16* for population-wide screening and *Table S17* for targeted re-screening

Consistent with the categorical NRI analyses above, we observed statistically significant incremental improvements in primary prevention of CVD when applying all seven alternative models in population-wide screening, i.e. the primary care population eligible for screening in the UK (*Figure 5A*, Supplementary data online, *Table S16*). For all seven alternative models, we observed significant increases in N_high-risk_ and CVD_high-risk_, with concomitant increases in CVD_prevented_ and decreases in NNS. CVD_prevented_ per 100 000 screened increased from 208 with SCORE2 alone, to 254 with addition of PRSs (ΔCVD_prevented_: 47; 95% CI: 39, 55; *P*-value: 1 × 10^−31^), to 301 with addition of NMR scores (ΔCVD_prevented_: 92; 95% CI: 83, 101; *P*-value: 1 × 10^−88^), to 317 with addition of 11 clinical biomarkers (ΔCVD_prevented_: 118; 95% CI: 108, 128; *P*-value: 9 × 10^−122^), and to 376 with addition of PRSs, NMR scores, and clinical biomarkers combined (ΔCVD_prevented_: 177; 95% CI: 165, 189; *P*-value: 4 × 10^−192^) (see Supplementary data online, *Table S16*). Importantly, our modelling indicated no statistically significant change in NNT, which was constant at 22 (*Figure 5A*, Supplementary data online, *Table S16*).

Targeted re-screening of the population classified as medium risk by SCORE2 (36 054 per 100 000) with NMR scores, clinical biomarkers, and/or PRSs yielded slightly stronger incremental improvements in primary prevention of CVD as compared to the above population-wide screening with SCORE2 alone. This was accompanied by a small but statistically significant increase in NNT (ΔNNT: 1) when re-screening with NMR scores, clinical biomarkers, or PRSs separately (*Figure 5B*, Supplementary data online, *Table S17*). If treatment was recommended for the same number of additional people simply prioritized by SCORE2, targeted re-screening yielded stronger incremental improvements in CVD_prevented_ (ΔCVD_prevented_ range: 16, 59) and significantly lower NNT (ΔNNT range: −1, −3) (*Figure 5B*, Supplementary data online, *Table S17*). Targeted re-screening with NMR scores, clinical biomarkers, and PRSs combined increased CVD_prevented_ per 100 000 from 229 to 413 (ΔCVD_prevented_: 184; 95% CI: 174, 194; *P*-value: 4 × 10^−273^) whereas prioritizing the same number of additional people for treatment with SCORE2 increased CVD_prevented_ to 354 (ΔCVD_prevented_: 125; 95% CI: 117, 133; *P*-value: 3 × 10^−202^). With targeted re-screening, NNT remained constant at 22 (ΔNNT: 0.24; 95% CI: −0.42, 0.89; *P*-value: 0.48) whereas prioritizing the same number of additional people for treatment with SCORE2 increased NNT to 26 (ΔNNT: 3.29; 95% CI: 2.69, 3.88; *P*-value: 2 × 10^−27^).

Results were similar when analysing the discovery and replication cohorts separately (see Supplementary data online, *Figure S6*, Supplementary data online, *Table S18* and *S19*) and when using a more narrowly defined CVD definition (see Supplementary data online, *Figure S7*, Supplementary data online, *Tables S18* and *S19*). Results were also similar in both males and females, including expected differences in magnitudes arising from differences in CVD incidence (9025 per 100 000 males screened vs. 4447 per 100 000 females screened; Supplementary data online, *Figure S8* and *S9*, Supplementary data online, *Tables S18* and *S19*). Improvements in primary prevention of CVD when using QRISK3 and NICE 2023 guideline-recommended risk thresholds were smaller than those observed when using SCORE2 (see Supplementary data online, *Figure S10*, Supplementary data online, *Tables S18* and *S19*) as expected due to the dominating influence of age on risk stratification observed above (see Supplementary data online, *Table S15*). Notably, the total number of statins prescribed (*i.e.* N_high-risk_) and NNT were significantly higher with QRISK3 (N_high-risk_: 28 005; NNT: 34) than SCORE2 (N_high-risk_: 4669; NNT: 22).

## Discussion

Determining the added value of biomarkers beyond total and HDL cholesterol for 10-year CVD risk prediction is an area of interest for enhancing CVD prevention.^3^ Here, we investigated whether 10-year CVD risk prediction in UK Biobank participants eligible for screening could be improved, in comparison to the currently recommended SCORE2.^3,4^

We found statistically significant improvements in 10-year CVD risk prediction from both PRS and 66 of 277 biomarkers quantified either individually by clinical chemistry assays or simultaneously by plasma NMR spectroscopy. Combining information from multiple biomarkers more than doubled the gain in observed predictive performance (ΔC-index) as compared to any single biomarker. Scores of established clinical biomarkers, NMR metabolomic biomarkers, and PRSs offered largely orthogonal information, with clinical biomarker scores offering the greatest improvement in C-index when added to SCORE2. As expected, the largest improvements in predictive performance were obtained when adding all biomarkers to SCORE2.

When added to SCORE2, the clinical biomarkers yielding the strongest improvements in 10-year CVD risk prediction were cystatin-C, Lp(a), Albumin, CRP, and vitamin D. Each of these are well-known biomarkers of CVD risk,^42–46^ and for CRP and Lp(a), biomarkers of long-standing interest for CVD risk prediction.^3,20,47^ A key CVD risk pathway intersected by all five biomarkers is inflammation,^48–52^ which is a well-studied target in CVD prediction and prevention research.^53^ Likewise, the strongest contributors to the NMR scores were albumin and glycoprotein acetyls (GlycA), an NMR signal quantifying the levels of multiple inflammatory proteins^54,55^ and a stronger biomarker of chronic inflammation than CRP,^56^ which has been associated with CVD risk in multiple studies.^57–59^

Risk prediction models including clinical biomarkers, NMR metabolomic biomarker scores, and/or PRSs also improved risk stratification of future CVD events when using risk thresholds recommended for clinical decision making by the ESC 2021 guidelines for CVD prevention. Clinical biomarkers and NMR scores improved net case reclassification to a greater extent than PRSs (11.63%, 8.85%, and 4.33% respectively); however, when combined, clinical biomarkers, NMR scores, and PRSs improved net case reclassification by 16.66%. These results highlight the complementary nature of the information capture by PRSs and circulating biomarkers. While PRSs capture the lifetime risks due to genetics,^10–12^ circulating biomarkers capture part of the dynamic component of risk conferred by lifestyle and environment,^26^ which act on that genetic background.

When modelling the potential benefits for primary prevention of CVD in the wider UK population eligible for screening, we found adding clinical biomarkers, NMR scores, and/or PRSs to SCORE2 significantly increased the proportion of the population recommended for statin initiation (following the ESC 2021 guidelines for risk factor treatment for CVD prevention^3^) and who would subsequently experience a CVD event. Importantly, the number of statins prescribed per CVD event prevented stayed constant.

To increase its efficiency, we also modelled the potential benefits of targeted follow-up screening in those at medium risk, for whom the ESC 2021 guidelines suggest considering, but do not explicitly recommend, risk factor treatment.^3^ We found that, when compared to population-wide screening, targeted re-screening with combinations of clinical biomarkers, metabolomic biomarkers, and/or PRSs would yield similar improvements in the number of CVD events prevented while essentially maintaining the number of statins prescribed per CVD event prevented. We also found that targeted re-screening would be more efficient than simply using SCORE2 to prioritize the same number of additional people for treatment, estimating that targeted re-screening would lead to both more CVD events prevented and a lower number of statins prescribed per event prevented. We estimated that targeted re-screening with clinical biomarkers, NMR metabolomic biomarkers, and PRSs combined would increase the number of CVD events prevented per 100 000 screened from 229 to 413 while maintaining the number of statins prescribed per event prevented at 22, whereas prioritizing the same number of additional people for treatment with SCORE2 would lead to a smaller increase in the number of CVD events prevented to 354 while also increasing the number of statins prescribed per event prevented to 26.

This study has several limitations. The set of biomarkers assessed in this study was limited to those that were measured in UK Biobank, which did not include some important biomarkers of cardiovascular risk, such as natriuretic peptides (e.g. NT-proBNP) and troponins.^60,61^ While we were able to replicate our results using an independent set of UK Biobank participants, we were unable to assess generalizability across populations due to lack of concomitant data on metabolomics, genetics and clinical biomarkers in studies with large numbers of incident CVD events. Notably, different regions of Europe vary in CVD risk profiles^4^ and thus may have different distributions of some biomarkers. Further, our study comprised almost entirely (>95%) European ancestries and thus may not generalize to other ancestry groups which have different risk profiles.^62^ Importantly, development of SCORE2 required information on risk factor distributions from a range of cohorts to calibrate risk factor distributions and their estimates for 10-year CVD risk,^4^ which would also need to be the case for any extensions to SCORE2 with additional biomarkers or PRSs. Our estimates of incremental improvement for primary prevention of CVD relied on a demographic standardization procedure with multiple assumptions and sources of competing biases. For example, incremental benefits may be underestimated due to the healthy ascertainment bias of UK Biobank,^30^ but at the same time may be overestimated as expected 10-year CVD incidence rates were estimated in CVD- and statin- free primary care patients,^15^ whereas the age- and sex- demographics were obtained from the general UK population^40^ and thus could not account for screening eligibility with increasing age. However, whilst these may impact the precise magnitude of benefits for primary prevention of CVD, they do not impact the relative incremental benefits when comparing models developed in this study to SCORE2 alone.

Across all our analyses, incremental gains in CVD prediction, risk stratification, and screening efficacy were larger when combining information from 11 established clinical biomarkers compared to using NMR scores or PRS. However, caution should be exercised in interpretation as this additional incremental gain comes with a trade-off of increased complexity and potentially cost; requiring quantification via multiple different assay types, providers, and in some cases, sample preparation techniques.^32^ In contrast, PRS and NMR scores each require a single assay, with the latter including the total and HDL cholesterol measures required for SCORE2.^23^ Further, it should be noted that the stability across cohorts and populations of the NMR and clinical biomarker scores across utilized here have not been as extensively assessed as PRS, thus relative performance may vary.

Overall, this study represents the largest population health assessment of metabolomic and genomic biomarkers for CVD to date. While our findings suggest that there are potential gains for CVD risk prediction and prevention, there are obvious challenges for validating clinical utility and potential implementation. Commercial providers of NMR biomarkers and PRSs exist, yet fidelity, scale, and cost frequently mean that real world benefits are less than those estimated in prospective cohort studies. Nevertheless, our results indicate that current technologies that can scale to populations (e.g. NMR metabolomics and genomics) have the capacity to improve CVD risk prediction. However, further studies are needed to evaluate the efficacy and cost-effectiveness of additional established clinical biomarkers, NMR scores, and/or PRSs for improving 10-year CVD risk prediction in these settings. For the goal of primordial prevention, further studies are also needed to investigate the potential for circulating biomarkers for CVD risk prediction in younger adults.^63^

In conclusion, our results indicate that incorporating biomarker scores into 10-year CVD risk prediction could enhance prediction of first-onset CVD. We further add to the growing body of evidence that PRSs can be used to enhance CVD risk prediction over conventional risk factors^12,15^ and show that improvements in 10-year CVD risk prediction from PRSs are orthogonal to, and can be combined with, scores of clinical or metabolomic biomarkers. Applied at scale, integrating additional NMR metabolomic, polygenic, and established clinical biomarkers with SCORE2 may have population health benefit.

## Supplementary Material

ehaf947_Supplementary_Data

## Declarations

### Data Availability

All data described are available through UK Biobank subject to approval from the UK Biobank access committee. See https://www.ukbiobank.ac.uk/enable-your-research/apply-for-access for further details.

### Code availability

Code underlying this paper is available at https://github.com/sritchie73/UKB_NMR_CVD_prediction/. This repository and specific release for this paper are permanently archived by Zenodo at https://doi.org/10.5281/zenodo.16895329.
